# A Rare Case of Neurogenic Pulmonary Edema Following High-voltage Electrical Injury

**DOI:** 10.5005/jp-journals-10071-23226

**Published:** 2019-08

**Authors:** Gopal Chawla, Naveen Dutt, Ramniwas, Nishant Chauhan, Vinod Sharma

**Affiliations:** 1-5 Department of Pulmonary Medicine, AIIMS, Jodhpur, Rajasthan, India

**Keywords:** Electric shock, Lung ultrasound score, Pulmonary edema

## Abstract

Electrical injuries though infrequent, are potentially devastating form of injuries which are associated with high morbidity and mortality. The severity of the injury depends upon intensity of the electrical current which is determined by the voltage and the resistance offered by the victim. These injuries vary from trivial burns to death. There have been few reports about pulmonary injuries due to electrical current but none mentioning neurogenic pulmonary edema (NPE). Here we report a young boy who when exposed to high voltage current developed neurogenic pulmonary edema and was successfully managed. Though there is no specific protocol for electrical injury but identifying the organs involved along with type of disease facilitates the management.

**How to cite this article:**

Chawla G, Dutt N, Ramniwas, Chauhan N, Sharma V. A Rare Case of Neurogenic Pulmonary Edema Following High-voltage Electrical Injury. Indian J Crit Care Med 2019;23(8):384–386.

## INTRODUCTION

Electrical injury should be managed as a multisystem injury as there is virtually no organ that is protected against it. Type and extent of an electrical injury depends on the intensity (amperage) of the electric current which is dependent on voltage of current and resistance offered by body. The same voltage will generate a different current causing a different degree of damage because resistance varies significantly between various tissues^1^ Here we present an unusual case of neurogenic pulmonary edema developed after high-voltage shock and its successful management.

## CASE DESCRIPTION

**Figs 1A to E:**
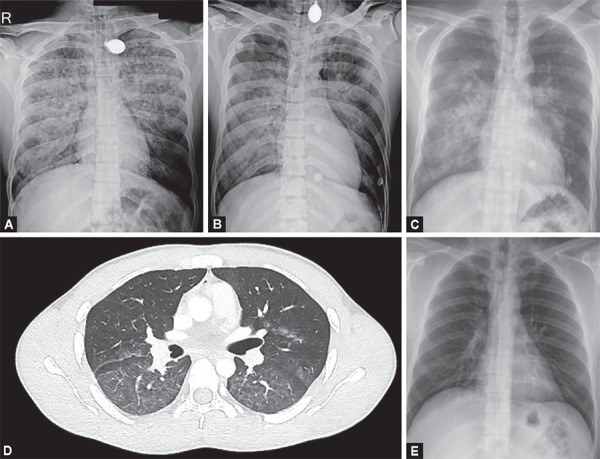
(A) Initial chest X-ray showing pulmonary edema; (B) Chest X-ray after 24 hours; (C) Chest X-ray after extubation when patient is on NIV; (D) HRCT showing diffuse GGO; (E) Chest X-ray after 7 days showing complete resolution

**Fig. 2:**
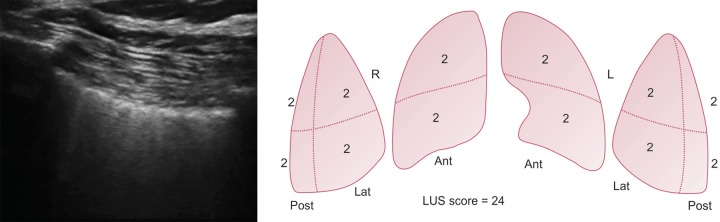
Ultrasound showing multiple B lines suggestive of interstitial syndrome with calculation of lung ultrasound score

A 23-year-old man was brought to emergency with alleged history of electrical injury by high voltage current when high voltage electric cable (440 V) broke and came in contact with metal wire over which he was drying his clothes. He presented to the emergency department within 5 minutes of the contact with the current. He was not breathing but radial pulse was palpable. On examination patient had entry wound in right palm and exit wound in left hand. ECG showed first degree atrio-ventricular block. Chest X-ray showed diffuse heterogeneous alveolar opacity suggestive of pulmonary edema (Fig. 1). On performing screening echo biventricular function appeared normal with no pericardial effusion. USG chest showed multiple bilateral B lines with continuous pleura, with an initial lung ultrasound score^2^ of 24 (Fig. 2). Blood gas analysis showed type 2 respiratory failure. Patient had normal electrolytes, CPK MB, NT-pro BNP, renal and liver function test.

Patient was intubated and started on mechanical ventilation. Endotracheal tube aspirate had pink frothy to white secretions. Patient was shifted to the ICU as he was not able to maintain saturation even with 100% FiO_2_. Blood gas analyses showed severe hypoxemia (PaO_2_/FiO_2_ 62.4) and managed with low tidal volume and high PEEP mechanical ventilation settings. Radiology of extremities and spine were normal. Noncontrast CT chest showed ground glass opacities in central lung in the bilateral upper, right middle lobe and superior segments of bilateral lower lobes with subtle interstitial thickening.

Patient was given adequate fluid resuscitation with normal saline infusion at a rate of 1.5 L/hour, aimed to maintain a urine output of 200–300 mL/hour. This was in anticipation of expected rhabdomyolysis which is expected in post-electric shock patients and may result in acute kidney injury.^3^ Pulmonary edema was initially managed with high PEEP of 16 and low tidal volume of 300 mL, FiO_2_ of 100%, respiratory rate of 26 to maintain minute ventilation of 5 mL/kg. The patient was kept sedated and paralysed initially, while gradually titrating FiO_2_ and PEEP. His LUS score improved from 24 to 16 within 24 hours. His FiO_2_ requirement decreased to 40%. Paralysis was stopped after initial 6 hours and sedation was discontinued the next day. He was given spontaneous breathing trial and after a successful SBT (spontaneous breathing trial) he was extubated on 3rd day over CPAP to prevent complications of delayed extubation. He was further weaned off from CPAP over the next 24 hours. During the course he had elevated serum creatinine kinase which increased for first 2 days to 300 and then started decreasing and normalized to 112 on the 5th day post injury. Chest radiograph performed on day 7 showed complete resolution of the opacities, and the patient was discharged.

## DISCUSSION

Our case had electrical injury while he was drying his wet clothes on a metal wire and high voltage (440 V) electric cable broke and came in contact with that wire. As sweat decreases skin resistance by 1000Ω, wet skin in our case offered no resistance at all and caused injury with maximum intensity possible with that voltage.^4^

Another important consideration is pathway of the current through the body (from the entry to the exit point) which determines the number of organs that get affected. Our patient had entry wound in right hand and exit in left hand forming a horizontal pathway from hand to hand which spared the brain but still could be fatal due to involvement of the heart, respiratory muscles, or spinal cord.^5^

Respiratory arrest is recognized as one of the important causes of acute death in high voltage electrical injuries even without specific injuries to the lungs or the airways. It is usually the result of direct injury to the respiratory control centre leading to cessation of respiration, or due to suffocation caused by tetanic contractions of the respiratory muscles, which occurs when the thorax is involved in the current pathway. Direct injury to the spinal cord in the form of transection at the C4–C8 level can occur with a hand-to hand current flow which often leads to “locking-on” phenomenon causing prevention of victim's hand separating from the electrical source. Autonomic instability causing hypertension and peripheral vasospasm have also been described in electrical injuries and are believed to result from massive release of catecholamine.^3,6,7^

There have been only a few case reports of electricity-associated lung injuries. The first case presented as acute cardiogenic pulmonary edema that followed electricity-induced ventricular fibrillation and cardiopulmonary resuscitation.^8^ While in other focal lung injury has been reported in an electrician after a low-voltage shock (380 V) which was complicated by respiratory arrest and subsequent cerebral edema. An area of consolidation was seen in right lower lobe, with no additional respiratory complications.^9^ Two cases were reported due to high-voltage electric current (≥1000 V), having focal consolidation on lung imaging, and were managed with surgical resection.^10,11^

Our case presented within a short duration of an accidental electrical injury and expectoration of pinkish frothy secretions from endotracheal tube along with chest X-ray and USG findings were suggestive of pulmonary edema. Cardiac dysfunction was ruled out by normal cardiac enzymes and echocardiography. In view of the rapidity of the onset of symptoms, the possibility of neurogenic pulmonary edema was considered.

Enhanced secretion of catecholamines from peripheral sympathetic nerve endings (blast theory or catecholamine surge) result in peripheral vasoconstriction causing an increase in systemic vascular resistance and subsequently an increase in systemic BP together with the augmentation of central blood volume and a reduction in the compliance of the LV. These changes are followed by the constriction of the pulmonary veins which lead to increase in pulmonary capillary hydrostatic pressure and later leading to damage of alveolar wall and leakage of fluid into the interstitium and intra-alveolar space along with hemorrhage resulting in the typical picture of neurogenic pulmonary edema.^12–14^ while Smith et al. observed that in 7 out of 12 cases it is the increased pulmonary hydrostatic pressure which causes neurogenic pulmonary edema and not the increased lung capillary permeability.^15^

## CONCLUSION

Electrical injury is often life threatening, detailed history regarding type of electric source and surroundings often helps in understanding the pathway of current thus helping in determining the organs involved. Pulmonary enema following electric injury can be cardiogenic or noncardiogenic. The management primarily consists of supportive care and adequate oxygenation in the form of either noninvasive or mechanical ventilation. This condition has a high risk of mortality if left unrecognised and untreated and therefore must be kept in mind while managing a patient with electrical injury.

## References

[1] Cooper MA. (1995 Sep;). Emergent care of lightning and electrical injuries.. Semin Neurol..

[2] Via G,, Storti E,, Gulati G,, Neri L,, Mojoli F,, Braschi A. (2012 Nov;). Lung ultrasound in the ICU: from diagnostic instrument to respiratory monitoring tool.. Minerva Anestesiol..

[3] Brumback RA,, Feeback DL,, Leech RW. (1995 Dec;). Rhabdomyolysis following electrical injury.. Semin Neurol..

[4] Hunt J,, Mason A,, Masterson T,, Pruitt B, (1976 May 1;). The pathophysiology of acute electric injuries.. The Journal of Trauma: Injury, Infection, and Critical Care..

[5] Jain S,, Bandi V. (1999 Apr;). Electrical and lightning injuries.. Crit Care Clin..

[6] Varghese G,, Mani MM,, Redford JB. (1986 Jun;). Spinal cord injuries following electrical accidents.. Paraplegia..

[7] Kleinschmidt-DeMasters BK. (1995 Dec;). Neuropathology of lightning-strike injuries.. Semin Neurol..

[8] Schein RMH,, Kett DH,, Marchena EJD,, Sprung CL. (1990 May 1;). Pulmonary Edema Associated with Electrical Injury.. Chest..

[9] Karamanli H,, Akgedik R. (2017 Sep 3;). Lung damage due to low-voltage electrical injury.. Acta Clinica Belgica..

[10] Masanès MJ,, Gourbière E,, Prudent J,, Lioret N,, Febvre M,, Prévot S, (2000 Nov 1;). A high voltage electrical burn of lung parenchyma.. Burns..

[11] Schleich AR,, Schweiger H,, Becsey A,, Cruse CW. (2010 Aug;). Survival after severe intrathoracic electrical injury.. Burns..

[12] Sarı MY,, Yıldızdaş RD,, Yükselmiş U,, Horoz ÖÖ. (2015 Dec 1;). Our patients followed up with a diagnosis of neurogenic pulmonary edema.. Turk Pediatri Ars..

[13] Simon RP. (1993 May;). Neurogenic pulmonary edema.. Neurol Clin..

[14] Fontes RBV,, Aguiar PH,, Zanetti MV,, Andrade F,, Mandel M,, Teixeira MJ. (2003 Apr;). Acute neurogenic pulmonary edema: case reports and literature review.. J Neurosurg Anesthesiol..

[15] Smith WS,, Matthay MA. (1997 May;). Evidence for a hydrostatic mechanism in human neurogenic pulmonary edema.. Chest..

